# Effect of culture medium, host strain and oxygen transfer on recombinant Fab antibody fragment yield and leakage to medium in shaken *E. coli* cultures

**DOI:** 10.1186/1475-2859-12-73

**Published:** 2013-07-29

**Authors:** Kaisa Ukkonen, Johanna Veijola, Antti Vasala, Peter Neubauer

**Affiliations:** 1 Department of Process and Environmental Engineering, Bioprocess Engineering Laboratory, University of Oulu, Oulu, Finland; 2 BioSilta Oy, Oulu, Finland; 3 Department of Physiology, Institute of Biomedicine, University of Oulu, Oulu, Finland; 4 Department of Biotechnology, Laboratory of Bioprocess Engineering, Technische Universität Berlin, Berlin, Germany

**Keywords:** Fab fragment, Periplasmic expression, Oxygen transfer, Fed-batch, Autoinduction

## Abstract

**Background:**

Fab antibody fragments in *E. coli* are usually directed to the oxidizing periplasmic space for correct folding. From periplasm Fab fragments may further leak into extracellular medium. Information on the cultivation parameters affecting this leakage is scarce, and the unpredictable nature of Fab leakage is problematic regarding consistent product recovery. To elucidate the effects of cultivation conditions, we investigated Fab expression and accumulation into either periplasm or medium in *E. coli* K-12 and *E. coli* BL21 when grown in different types of media and under different aeration conditions.

**Results:**

Small-scale Fab expression demonstrated significant differences in yield and ratio of periplasmic to extracellular Fab between different culture media and host strains. Expression in a medium with fed-batch-like glucose feeding provided highest total and extracellular yields in both strains. Unexpectedly, cultivation in baffled shake flasks at 150 rpm shaking speed resulted in higher yield and accumulation of Fabs into culture medium as compared to cultivation at 250 rpm. In the fed-batch medium, extracellular fraction in *E. coli* K-12 increased from 2-17% of total Fab at 250 rpm up to 75% at 150 rpm. This was partly due to increased lysis, but also leakage from intact cells increased at the lower shaking speed. Total Fab yield in *E. coli* BL21 in glycerol-based autoinduction medium was 5 to 9-fold higher at the lower shaking speed, and the extracellular fraction increased from ≤ 10% to 20-90%. The effect of aeration on Fab localization was reproduced in multiwell plate by variation of culture volume.

**Conclusions:**

Yield and leakage of Fab fragments are dependent on expression strain, culture medium, aeration rate, and the combination of these parameters. Maximum productivity in fed-batch-like conditions and in autoinduction medium is achieved under sufficiently oxygen-limited conditions, and lower aeration also promotes increased Fab accumulation into extracellular medium. These findings have practical implications for screening applications and small-scale Fab production, and highlight the importance of maintaining consistent aeration conditions during scale-up to avoid changes in product yield and localization. On the other hand, the dependency of Fab leakage on cultivation conditions provides a practical way to manipulate Fab localization.

## Background

Fragments of immunoglobulin molecules are widely utilized in therapeutic and diagnostic applications as well as in basic research. Unlike full-length antibodies, these smaller fragments, such as the antigen binding fragments (Fab) and single-chain variable fragments (scFv), are small enough to be produced in *Escherichia coli*. However, the yields of correctly folded, functional antibody fragments in *E. coli* are often relatively low and dependent on the type and primary sequence of the fragment. Yields in the range of 10–20 mg functional Fab fragments per liter of culture are generally considered good in shake flask scale [1-3]. Major challenges in bacterial antibody fragment expression are the assembly of separately expressed light and heavy chain to constitute the functional heterodimer and formation of the four intra-chain and one inter-chain disulfide bond [4]. Since the disulfides cannot be efficiently formed in the reducing cytoplasm of *E. coli*, antibody fragments are most commonly supplemented with a signal sequence that directs them to the more oxidizing bacterial periplasm for correct folding. Folded fragments may further leak from the periplasm into the culture medium, from which purification can be accomplished without cell lysis [4]. An alternative strategy is to use redox mutant strains with more oxidizing cytoplasm for folding of the fragments in the *E. coli* cytoplasm [3,5-7], but these mutant strains tend to have poor growth that limits their capacity for protein production and scale-up to fermenter scale.

Previously described approaches to improve antibody fragment yields in *E. coli* have mostly focused on the optimization of the expression construct and the target fragment itself. For example, co-expression of different accessory proteins such as the cytoplasmic DnaKJE chaperone [8] or periplasmic dithiol-disulfide oxidoreductases and prolyl *cis-trans* isomerases [9] have been reported to increase yields of Fab and scFv fragments. Fusion to maltose-binding protein (MBP) has been shown to not only increase solubility of antibody fragments [10,11], but also enhance secretion from periplasm into the culture medium in secretory *E. coli* strains [10]. MBP fusion [12] as well as thioredoxin [13] and SUMO fusions [14] have also been reported to improve scFv yields in the cytoplasm of redox mutant strains. In some cases yield may also be increased by engineering the amino acid sequence in non-binding regions of the fragment to reduce its aggregation tendency [15].

A few reports exist on the optimization of culture medium and strain selection for antibody fragment production. Nadkarni et al. [1] compared defined media with different carbon sources and induction strategies, and found Studier’s lactose autoinduction medium to provide higher Fab yields than either glycerol-based defined medium with lactose induction or glucose-based defined medium with IPTG induction. The authors also compared two expression strains, BL21(DE3) and BL21(DE3)-RIL, although these strains differ from each other only regarding rare codon utilization but not regarding carbon metabolism. The effect of inducer on Fab expression has also been studied in *E. coli* K-12 RB791, in which highest Fab yields were obtained by induction with either a very low IPTG concentration or 2 g l^-1^ lactose using glycerol as the main carbon source [16]. Supplementation of culture medium with L-arginine and reduced glutathione [17] or sucrose [18] has been described as means to increase yields of functional scFvs. Glutathione was suggested to improve reshuffling of incorrectly formed disulfides, while the effect of sucrose was hypothesized to be due to osmotic enlargement of the periplasmic space and consequently enhanced folding of the product as a result of reduced local concentration. Cultivation temperature has been reported to influence the secretion into the culture medium so that at lower temperatures the product is more efficiently retained in the periplasm [18].

In this study we aim to investigate the effects of host strain, culture medium and aeration conditions on the production and extracellular leakage of Fab fragments in shaken *E. coli* cultures by the example of Fabs binding specifically to N-terminal pro-brain natriuretic peptide (NTproBNP), an important diagnostic marker of heart failure that can be detected from serum by an immunoassay applying the anti-NTproBNP Fabs [19]. Three different culture media were compared, all of them containing complex nutrients, but differing in their primary carbon source as well as in induction strategy. In the Super Broth medium, peptides, amino acids and sugars of yeast extract constitute the main carbon source during IPTG-induced expression. In Studier’s autoinduction medium [20], growth is first supported by glucose, and when glucose is exhausted protein expression is autoinduced by diauxic shift to lactose utilization, while glycerol is also coutilized as a major carbon source during expression. The third medium was the fed-batch-like EnBase® medium with IPTG induction. In this medium the primary carbon source, glucose, is gradually provided from a soluble polysaccharide by biocatalytic degradation [21,22]. The polysaccharide used in the current study is different from the previous reports in that it is also slowly utilized to some degree through the *E. coli* maltose-maltodextrin transport system (own unpublished results). The EnBase fed-batch-like medium has been successfully used for high-yield cytoplasmic expression of several non-disulfide bond containing proteins [22-27] as well as functional protein with multiple disulfide bonds [28,29], while in this study we apply this medium for the first time for periplasmic production of disulfide-containing proteins. We also compared two metabolically different *E. coli* strains regarding their Fab yield in the different growth media. Apart from differences in Fab yields, we also observed some peculiar effects on leakage of the Fabs into the culture medium depending on the type of medium, host strain, and aeration efficiency.

## Results

### Comparison of culture media in small scale

**Figure 1:**
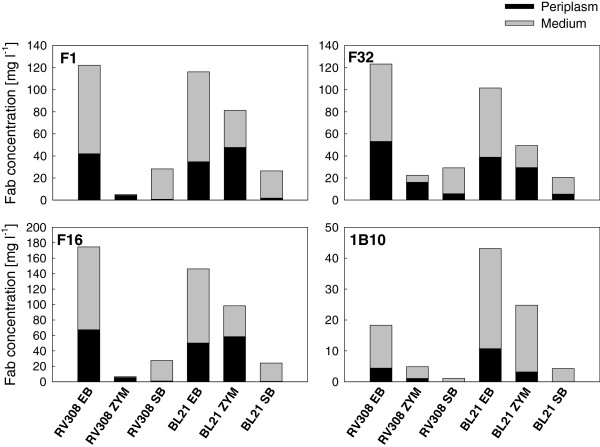
**Yields of Fab fragments in mg per liter of culture in different media.** Quantities of Fab fragments F1, F16, F32 and 1B10 were measured by antigen-binding ELISA from cell lysate (periplasmic fraction; in black) and broth supernatant (medium fraction; in grey). All fragments were expressed in *E. coli* RV308 and BL21 in 24 deep well plates. Samples were drawn for analysis at 24 h after induction in the fed-batch-like EnBase medium (EB), 19 h after induction in Super Broth (SB), and 19 h and 42 h after cultivation start in ZYM-5052 autoinduction medium (ZYM; 19 h data not shown, yields at 19 h were lower than at 42 h). The mean values of two independent replicate cultivations are shown.

Fab fragment expression in *E. coli* RV308 and *E. coli* BL21 was compared in three different media in 24 deep well plate (24dwp) cultures. Notable differences were observed in both the total yield and localization of the Fabs (Figure 1). The fed-batch medium provided highest total yields in both strains, and 60-75% of active product was found in the extracellular medium at 24 h after induction (Figure 1 and Table 1). In the autoinduction medium, all four fragments were produced at high concentrations in *E. coli* BL21(DE3), but for three of the fragments the proportion of extracellular product (40%) was lower than in the fed-batch medium. Low levels of Fab activity were detected also in *E. coli* RV308 when cultivated in the autoinduction medium, even if this strain is a Δ*(lac)X74* mutant and the expression must therefore be accounted to leakiness of the promoter. Fabs were most efficiently transported to extracellular medium when expressed in the Super Broth medium, in which 72-97% of product activity was measured in the extracellular fraction irrespective of fragment or host strain. However, the total Fab yields in Super Broth were much lower than in the other two media. Thus the small scale results suggest that the fed-batch medium is the most favorable medium for Fab production due to the high overall yield and efficient transport of the product to extracellular medium, as well as the robustness regarding strain type.

**Table 1:** Cell density, pH and percentage of extracellular Fab in different media in 24dwp

		**% of Fab in medium**	**OD**_**600**_			**pH**	
			**19 h**	**24 h**	**42 h**	**19 h**	**42 h**
RV308	EnBase	65.3 ± 2.6		21.7 ± 1.9	26.9 ± 2.7		7.07 ± 0.01
	ZYM-5052	6.8 ± 1.6	12.4 ± 3.7		14.1 ± 0.8		6.90 ± 0.02
	Super Broth	96.1 ± 2.4	13.8 ± 0.5			8.31 ± 0.18	
BL21(DE3)	EnBase	69.6 ± 2.6		19.8 ± 1.8	21.7 ± 6.5		6.70 ± 0.06
	ZYM-5052	39.8 ± 15.2	16.8 ± 5.1		17.5 ± 7.5		7.15 ± 0.02
	Super Broth	93.1 ± 1.9	8.5 ± 0.5			8.5 ± 0.04	

Cell density was determined by optical density measurements at 600 nm (OD_600_), and pH was measured from culture supernatant at room temperature. Mean and standard deviation of two independent replicate experiments are shown. In the fed-batch-like cultures (EnBase) OD_600_ is shown at 24 h (6 h from induction) and 42 h (25 h from induction). In ZYM-5052 autoinduction cultures OD_600_ is shown at 19 h and 42 h from cultivation start. In Super Broth cultures OD_600_ was measured after 19 h incubation in the inducing medium. pH was measured at the end of cultivation. The percentage of extracellular Fab represents the situation at the end of cultivation.

The main reason for higher product concentration in the fed-batch medium compared to the autoinduction medium appears to be higher cell density (cell density data for one representative Fab are shown in Table 1) rather than notably higher productivity per biomass. A reliable calculation of product per biomass was however not possible on the basis of OD_600_, since visual observation of DNA aggregates in the medium at 42 h indicated some degree of cell lysis especially in *E. coli* BL21(DE3) cultures. Lysis was apparently one of the reasons for Fab release from periplasm to medium in *E. coli* BL21(DE3), and possibly also in *E. coli* RV308.

Cultivation in Super Broth resulted in high final pH ranging from 8.0 to 8.5 (data for one representative Fab are shown in Table 1), while in the fed-batch and autoinduction media pH remained at a lower and more neutral range (6.6-7.2 depending on the clone and medium, except for the clones expressing Fab 1B10 which resulted in pH decrease to levels below 6.0; data not shown). The pH increase in Super Broth is in line with our earlier observations on pH development in complex media without added monosaccharide carbon sources [22,23], and likely limited both the final cell density and Fab yield.

### Medium composition, respiratory activity and Fab localization

**Figure 2:**
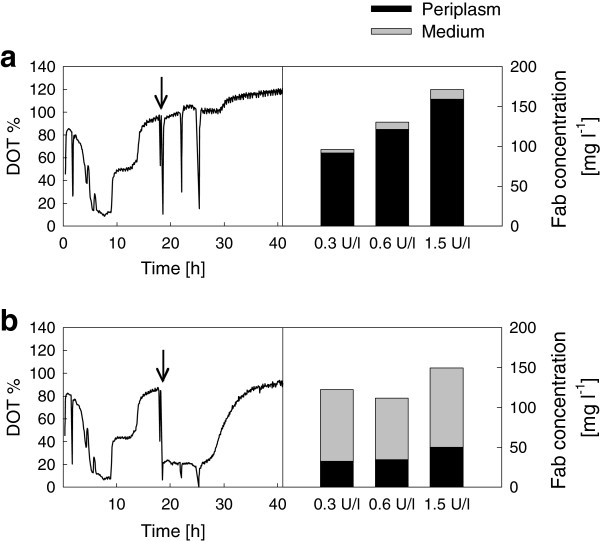
**Influence of fed-batch medium composition on oxygen saturation and Fab production.** Dissolved oxygen tension (DOT, % of saturation) was measured online during 24 microwell plate expression of Fab fragment F1 in *E. coli* RV308 in the fed-batch-like EnBase medium. Fab yields in mg per liter of culture were measured by antigen-binding ELISA from cell lysate (periplasmic fraction; in black) and broth supernatant (medium fraction; in grey) at 41 h. **a:** Cultivation without addition of nutrients at induction; **b:** Cultivation with addition of complex nutrients and 3 U l^-1^ biocatalyst at induction (18 h, indicated by arrows). In each case, Fab yields are shown for cultures with initial biocatalyst concentrations of 0.3, 0.6 and 1.5 U l^-1^. Representative DOT graphs are shown from the cultures with initially 0.6 U l^-1^ biocatalyst. DOT profiles with initial biocatalyst concentrations of 0.3 and 1.5 U l^-1^ were essentially similar to the graphs shown. Standard deviations for the ELISA analysis are shown in Additional file 1.

A separate small-scale cultivation was performed to study the influence of fed-batch medium composition on the dynamics of dissolved oxygen tension (DOT) during Fab fragment expression in *E. coli* RV308 (Figure 2). The pre-induction medium composition was kept constant, and modification was achieved by addition of more nutrients at the time of induction. Switch from initially unlimited growth to fed-batch-like limited growth took place at 9–10 h, and DOT at the time of induction (18 h) was 80-100% in all cultures. The cultures that did not receive complex nutrient supplementation and more glucose-releasing biocatalyst at induction maintained DOT at 100% after induction (Figure 2a). Addition of complex nutrients and more biocatalyst at induction (18 h) resulted in increased respiratory activity, and consequently DOT remained at a lower level (20-30%) for a period of 8–10 h after induction (Figure 2b). The increased oxygen consumption by addition of complex nutrients and increased glucose release was associated with high Fab activity in the extracellular medium (66-73% of total Fab activity, Figure 2b; see also in Additional file 1: Table S1b), while in the cultures with lower respiration and 100% oxygen saturation the product remained mostly in the periplasm (Figure 2a; see also in Additional file 1: Table S1a). pH was maintained between 7.0 and 7.5 in both cases (data not shown). Though the independent effects of DOT, growth rate and metabolic changes on Fab localization cannot be evaluated separately in this experiment, the results demonstrate that in the fed-batch medium the ratio of periplasmic and extracellular Fab can be drastically changed by modifying the availability of carbon and nitrogen substrates and consequently the respiratory rate after induction.

### Influence of shaking speed on Fab yield and localization

**Figure 3:**
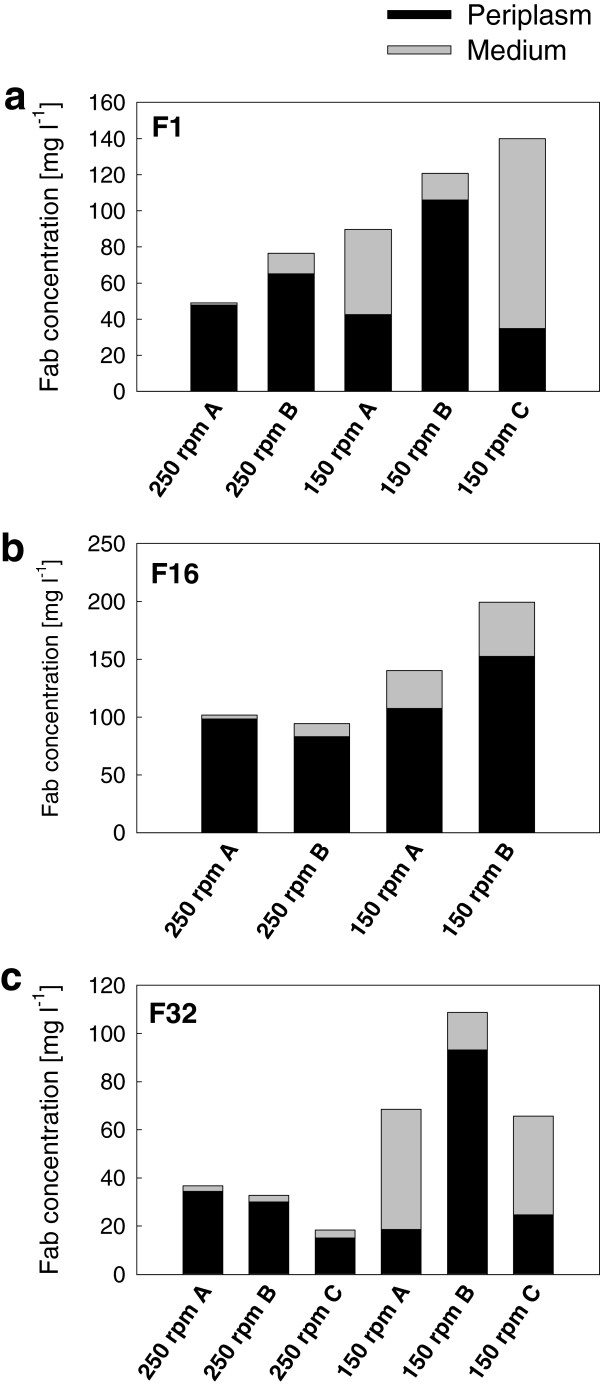
**Fab expression in *E. coli *RV308 in shake flask cultures.** Yields in mg per liter of culture for Fab fragments F1 **(a)**, F16 **(b)** and F32 **(c)** expressed in *E. coli* RV308 in the fed-batch-like EnBase medium in Ultra Yield shake flasks at two different shaking speeds (150 rpm and 250 rpm), as measured by antigen-binding ELISA from cell lysate (periplasmic fraction; in black) and broth supernatant (medium fraction; in grey). Samples were drawn 24 after induction. A-C on the horizontal axis refer to independent replicate experiments. Standard deviations for the ELISA analysis are shown in Additional file 2.

Expression of the Fab fragments in shake flask scale demonstrated that the yield and extracellular leakage can be influenced by modification of aeration efficiency via shaking speed. Cultures in the fed-batch medium were incubated at 250 rpm shaking speed in baffled Ultra Yield Flasks™ (UYF) up until induction, after which the speed was either reduced to 150 rpm (providing *k*_*L*_*a* ~200 h^-1^[30]) or kept at 250 rpm (providing *k*_*L*_*a* ~500 h^-1^[23]). Expression in *E. coli* RV308 at the lower shaking speed resulted consistently in higher yields of fragments F1, F16 and F32, even if there was some experiment-to-experiment variation in yield between replicates (Figure 3). Reduction of shaking speed also resulted in significant changes in Fab localization so that most of the Fab activity was detected in the medium as opposed to the efficient periplasmic retention of Fab at 250 rpm (Figure 3). This effect was observed for F1 and F32 in two out of three replicate experiments (A and C in Figure 3) at 150 rpm, while in the third experiment (B) there was much less leakage into the medium. Despite this inconsistency, which is may be caused by differences in oxygen uptake rate (OUR) between the replicates, the data suggest that there is a tendency towards higher extracellular Fab accumulation under conditions of lower oxygen supply. The extracellular proportion of fragment F16 was lower than for the other fragments, but consistently higher at 150 rpm compared to 250 rpm. Unlike the other three fragments, 1B10 leaked efficiently into the medium already at 250 rpm (data for 1B10 is shown in Additional file 2: Table S2a), and hence no difference in leakage was observed at different shaking speeds.

The degree of cell lysis was estimated by total protein measurement from cell pellet and culture supernatant by Bradford assay. Comparison of the percentage of cell lysis (as estimated from the relative concentrations of total protein in the cell pellet and in the medium; see in Additional file 2: Table S2a for the lysis estimates) and the percentage of Fab found in the culture medium suggests that at 250 rpm the small amount of fragments F1, F16 and F32 detected in the medium was released by cell lysis and there was no notable leakage from intact cells. The higher extracellular Fab yield at 150 rpm was partly due to higher cell lysis, but as the percentage of lysis was much lower than the percentage of extracellular Fab it is apparent that there was also increased leakage from intact cells. Depending on the fragment, at least 20-40% of total functional Fab leaked into the medium without accompanying lysis at 150 rpm. The possibility that the reduction of extracellular Fab fraction at the higher shaking speed might result from Fab denaturation due to the very efficient and turbulent shaking was ruled out by demonstrating over 95% preservation of binding activity when Fab-containing cell-free broth was shaken at 250 rpm for 24 h (data not shown).

**Figure 4:**
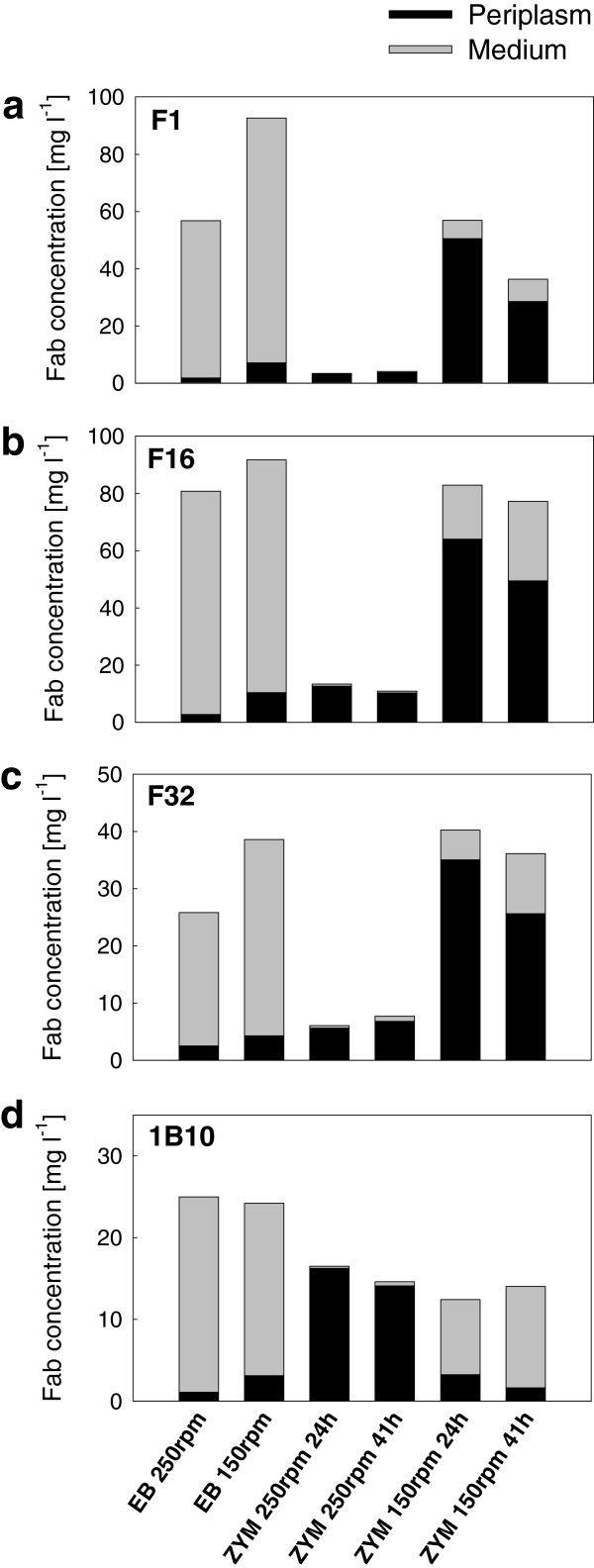
**Fab expression in *E. coli *BL21(DE3) in shake flask cultures.** Yields in mg per liter of culture for Fab fragments F1 **(a)**, F16 **(b)**, F32 **(c)** and 1B10 **(d)** expressed in *E. coli* BL21(DE3) in Ultra Yield shake flasks at two different shaking speeds (150 rpm and 250 rpm), as measured by antigen-binding ELISA from cell lysate (periplasmic fraction; in black) and broth supernatant (medium fraction; in grey). All fragments were expressed in the fed-batch-like EnBase medium (EB) and ZYM-5052 autoinduction medium (ZYM). Samples were drawn for analysis at 24 h after induction in EB, and 24 h and 41 h after cultivation start in ZYM. Standard deviations for the ELISA analysis are shown in Additional file 2.

Similar effect of shaking speed on yield and localization was observed for *E. coli* BL21(DE3) in the autoinduction medium, when cultures were performed in the UYF bottles with either 150 or 250 rpm shaking speed from the beginning. Total yields of F1, F16 and F32 were much higher at 150 rpm, and leakage of Fab into the medium also increased significantly at the lower shaking speed (Figure 4). The degree of lysis was low at both shaking speeds, but percentage of extracellular Fab increased from ≤ 10% to 20-30% of total Fab activity when the speed was reduced from 250 to 150 rpm (see in Additional file 2: Table S2b for the lysis estimates and percentages of extracellular Fab). Total yield of 1B10 in the autoinduction medium was not affected by the shaking speed (Figure 4), but extracellular Fab activity increased from 3 to 88% when speed was reduced to 150 rpm.

*E. coli* BL21(DE3) cultures in the fed-batch medium released Fabs very efficiently into the medium so that irrespective of shaking speed 87-97% of total Fab activity was detected in the medium after 24 h expression period (Figure 4; see also in Additional file 2: Table S2c for the percentages). Cell lysis was also substantial, typically 40-50% (lysis estimates are shown in Additional file 2: Table S2c). Total Fab yields were higher at the lower shaking speed, but the effect was less prominent than in the autoinduction medium.

Based on measurements at a few selected time points, pH was not significantly affected by the shaking speed in *E. coli* RV308 cultures (pH data are shown in Additional file 3: Tables S3a-S3c), and the differences in Fab yield and leakage are therefore not likely to be due to pH changes. In *E. coli* BL21(DE3), pH in the fed-batch medium was lower at the lower shaking speed, while in the autoinduction medium lower shaking speed contributed to consistently ~0.4 units higher pH. The pH change in fed-batch medium had apparently no influence on the extracellular Fab ratio in *E. coli* BL21(DE3).

### Influence of culture volume on Fab yield and localization

**Figure 5:**
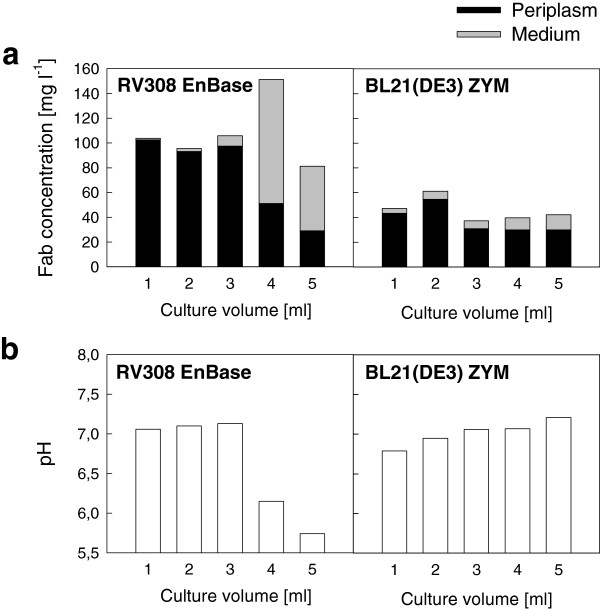
**Influence of culture volume in 24 deep well plates.** Yields of Fab fragment F1 in mg per liter of culture **(a)** and culture pH **(b)** in 24 deep well plate cultivations with varying broth volume (1–5 ml). Fab quantities were measured by antigen-binding ELISA from cell lysate (periplasmic fraction; in black) and broth supernatant (medium fraction; in grey). Fab was expressed in *E. coli* RV308 in the fed-batch-like EnBase medium, and in *E. coli* BL21(DE3) in ZYM-5052 autoinduction medium (ZYM). Samples were drawn for analysis at 24 h after induction in EnBase and 42 h after cultivation start in ZYM. Standard deviations for the ELISA analysis are shown in Additional file 4.

The finding that a change in shaking speed could so drastically influence Fab localization was unexpected, and we wanted to see whether this effect could be reproduced by modification of aeration efficiency via the culture surface to volume ratio. This was studied by varying the culture volume between 1 and 5 ml in the wells of a 24dwp. The results with Fab F1 expressed in *E. coli* RV308 in the fed-batch medium demonstrated significantly increased leakage into the extracellular medium with increasing culture volume (Figure 5a). The threshold was between 3 ml and 4 ml so that at 3 ml 92% of total Fab activity was retained in the periplasm, while at 4 ml 66% of Fab activity was found in the culture medium (the percentages of extracellular Fab are shown in Additional file 4: Table S4). When culture volume was further increased to 5 ml the total yield was reduced and anaerobic metabolism was indicated by a low pH (Figure 5b). These results with *E. coli* RV308 were reproduced in an independent repetition of the experiment. The data demonstrate that Fab localization may be drastically changed by a relatively small change in aeration efficiency, such as increase of culture volume by one third.

In *E. coli* BL21(DE3) cultures in the autoinduction medium the influence of culture volume on total Fab yield was minor (Figure 5a). It is likely that even at the lowest volume (1 ml) oxygen supply was below the threshold that caused significant productivity loss in the shake flask cultures at high shaking speed. Increasing culture volume contributed to gradual increase in the extracellular Fab fraction from 8% in 1 ml culture up to 28% in 5 ml culture (Figure 5a; see also in Additional file 4: Table S4 for the percentages of extracellular Fab). As *E. coli* cannot grow anaerobically on glycerol, no acidification was observed in the autoinduction medium with increasing severity of oxygen limitation (Figure 5b).

### Timeline of Fab leakage

**Figure 6:**
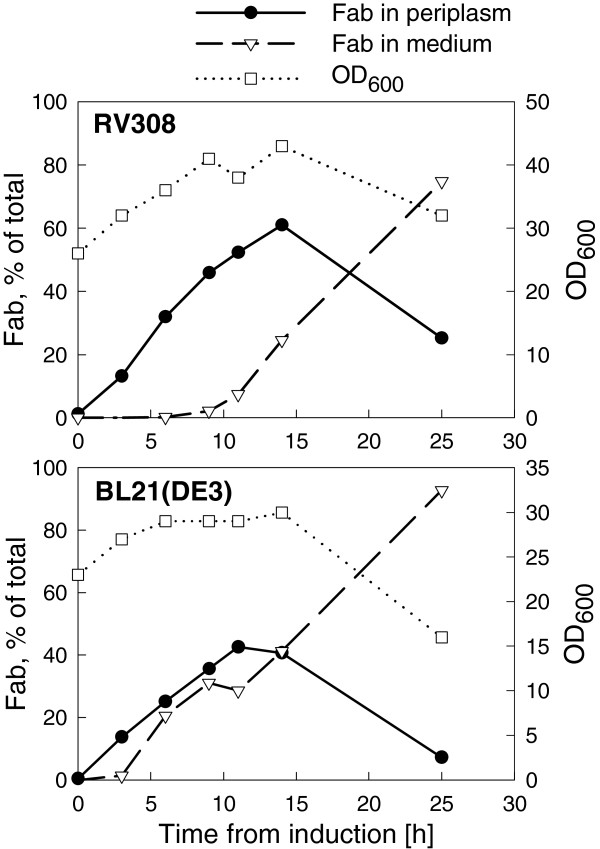
**Dynamics of Fab accumulation into periplasm and extracellular medium.** Fab fragment F1 was expressed in *E. coli* RV308 and BL21(DE3) in the fed-batch medium and Ultra Yield shake flasks at 150 rpm shaking. Fab quantities in cell lysate (periplasmic fraction; solid line with black circle) and broth supernatant (medium fraction; dashed line with triangle) were measured by antigen-binding ELISA, and are shown at different time points as percentage of the final Fab yield (left vertical axis). Cell density was recorded by OD_600_ readings (dotted line with square), which are shown on the right vertical axis.

To get a more detailed insight into the Fab release from periplasm to medium and the role of cell lysis in this, Fab accumulation and OD_600_ profiles were recorded from 150 rpm shake flask cultures in the fed-batch medium with both expression strains. Fragment F1 was expressed as the representative fragment. Fab accumulation into the medium started at approximately 9 h and 5 h after induction in *E. coli* RV308 and *E. coli* BL21(DE3), respectively (Figure 6). At the same time, Fab activity in the periplasm and OD_600_ were both still increasing, which indicates that the culture was not yet in stationary phase and not susceptible to cell lysis. At 14 h after induction, the proportion of extracellular Fab of total Fab activity at the time was 30% in RV308 and 50% in BL21(DE3), which can be accounted to lysis-independent leakage. When cultivation was continued into stationary phase (past 14 h from induction), part of the cells lysed and released more Fab into the medium, as indicated by a reduction in OD_600_. In the end, 75% and 92% of total Fab activity was found in the medium in RV308 and BL21(DE3), respectively. Based on OD_600_, the degree of lysis between 14 h and 25 h was 25% in RV308 and 46% in BL21(DE3). However, decrease in OD_600_ may also be partly due to shrinkage of cell size as the cells switch from active growth phase to stationary phase [31], and hence the degree of lysis may be slightly overestimated from the OD_600_ data. Assuming 25% lysis in RV308 after 14 h, the maximum amount of Fab released by lysis is 0.25 × (total Fab activity at 25 h – extracellular Fab activity at 14 h). Hence it is calculated that during the 25 h expression period at least 55% of total active Fab leaked into the medium without accompanying cell lysis. Correspondingly, the percentage of Fab leakage without lysis is estimated to be at least 65% of total Fab in BL21(DE3). The data demonstrate that Fab leakage in the fed-batch medium begins several hours before significant lysis, and thus it is possible to harvest extracellular Fab in the absence of cytoplasmic proteins by optimizing the harvest time.

Combined with the data on oxygen consumption (Figure 2), though from a different cultivation, the pattern of Fab accumulation (Figure 6) suggests that the leakage of Fab in *E. coli* RV308 starts around the time when respiratory activity of the culture decreases and oxygen level increases. DOT recording in small-scale *E. coli* BL21(DE3) expression culture (data not shown) indicated that in this strain a similar decrease in oxygen consumption takes place at 4–5 h after induction, which also coincides the start of Fab accumulation into the medium.

## Discussion

The finding that total Fab yields were reduced by high aeration was unexpected and contradictory to our earlier results with cytoplasmically expressed recombinant proteins in the fed-batch medium [23]. The largest effect of aeration on total Fab yield was observed in the autoinduction medium, in which total yield increased by 5 to 9-fold when shaker speed was reduced from 250 to 150 rpm. This is consistent with an earlier report by Blommel et al. [32], who demonstrated that protein expression in autoinduction media is highly dependent on the oxygenation state of the culture so that under oxygen limited conditions lactose consumption is preferred over consumption of glycerol, which in turn promotes earlier induction and higher total yield of the recombinant protein. The sensitivity of lactose and glycerol utilization patterns to oxygen availability could explain the adverse effect of high aeration on Fab expression in the autoinduction medium, but the reason for yield reduction under high aeration in the fed-batch medium is not clear. It is known that increased DOT can cause oxidative damage to recombinant proteins and their expression [33], but further studies would be needed to elucidate whether the observed reduction in functional Fab yield is due to oxygenation-dependent changes in the host metabolism or in the oxidative folding of Fab fragments in the periplasm. Interestingly, it seems that high DOT might be less detrimental to Fab expression in the fed-batch medium when the complex nutrient supplementation at induction is excluded and the post-induction growth rate is lower.

It is commonly acknowledged that antibody fragments can leak from *E. coli* periplasm to culture medium [4,34], and that this leakage takes place especially during extended cultivation periods [35]. In this study we observed the accumulation of Fab fragments in extracellular medium to increase under conditions of lower oxygen availability. This effect was observed in *E. coli* RV308 in the fed-batch medium, and in *E. coli* BL21(DE3) in the autoinduction medium. Part of the increased release of Fab into the cultivation medium can be accounted to increased cell lysis, but also leakage without lysis appears to increase significantly when aeration efficiency is reduced. The increase in Fab leakage could be due to the direct influence of DOT during expression, or due to changes in growth rate as a result of reduced oxygen supply. Growth rate has been reported to modify the outer membrane protein and lipid composition, and consequently influence the efficiency of periplasmic protein leakage [36,37]. In the study of Bäcklund et al. [37], increased growth rate in glucose-limited fed-batch contributed to higher product leakage into the extracellular medium, while according to Shokri et al. [36] the influence of growth rate may not be linear as they observed maximum leakage at a growth rate of 0.3 h^-1^, below or above which leakage decreased significantly. Both cell lysis and leakage from intact cells were influenced by the growth rate, and these were accompanied by changes in outer membrane lipid composition so that maximum in unsaturated fatty acids and minimum in saturated fatty acids coincided with the maximum in protein leakage. Therefore, changes in growth rate may be at least part of the mechanism by which the modification of oxygen supply via shaking speed or surface to volume ratio influenced the Fab leakage in our study. Growth rate during Fab expression could also be a contributing factor to the differences in the ratio of periplasmic and extracellular Fab observed between the different growth media.

Aeration can also influence the membrane lipid composition independent of growth rate, as has been earlier shown in chemostat cultures [38]. Decrease in aeration rate was reported to result in a decrease in unsaturated fatty acids and increase in cyclopropane fatty acids. An earlier study also reported similar changes in response to lower aeration rate [39]. The effect of these changes on protein leakage was not studied, but since both the decrease in unsaturated fatty acids and the increase in cyclopropane acids are known to reduce membrane fluidity they can be expected to contribute to reduced protein leakage. This seems contradictory to our findings that showed increased leakage at lower aeration rate even when the effect of lysis was subtracted. On the other hand, our data suggest that the beginning Fab leakage may coincide with an increase in DOT after a period of low oxygen saturation. Such a sharp change in DOT level contributes to substantial changes in the relative abundance of several outer membrane proteins [40], and this reorganization of the membrane structure could promote higher membrane permeability and leakage of the periplasmic product. Alternatively, it could also be the cumulative accumulation of Fab in the periplasm that eventually initiates leakage due to diffusive pressure, and decrease in OUR could coincide this moment as a result of the stress of high Fab accumulation on the cell and consequent decrease in growth rate. Since reduced aeration generally contributed to higher total yield of Fab, the diffusive pressure would be higher under these conditions. Also the increased transport of recombinant product to periplasm might in itself reduce the ability of the cell to transport structural elements to the outer membrane [36], resulting in a more permeable membrane structure allowing for higher diffusive leakage after sufficient product accumulation in the periplasm. However, total Fab concentration and leakage were not always correlated. In some cases increased leakage was observed without accompanying increase in total yield, suggesting that the leakage is more dependent on other factors than the periplasmic Fab concentration. These most likely include changes in the outer membrane composition due to either direct or indirect effects of DOT.

Extracellular pH may also affect the membrane fatty acid composition and hence the leakage efficiency of periplasmic proteins. There seems to be a tendency towards higher percentage of unsaturated fatty acids and lower percentage of cyclopropane acids with increasing pH [39], which suggests that higher pH might promote higher membrane permeability. However, we observed that lower oxygen availability contributed to increased Fab leakage in *E. coli* RV308 also in the absence of notable pH change, while in *E. coli* BL21(DE3) a pH decrease caused by reduced oxygen supply in the fed-batch medium was not associated with changes in Fab localization. Moreover, reduced aeration efficiency had opposite effects on pH in the fed-batch and autoinduction media, whereas Fab leakage increased in both. Thus it seems that the effect of pH at least in the range of 6.4 to 7.4 is minor, if any, and aeration influences Fab leakage by other mechanisms.

While further studies would be needed to confirm the independent effects of DOT, growth rate and pH on Fab leakage, our findings about the changes in Fab yield and leakage in response to aeration efficiency and medium composition have important practical implications for Fab production in shaken cultures. It is usually most straightforward to purify Fab fragments directly from the culture medium, and hence the goal in Fab production is in most cases to maximize the extracellular yield. Based on our results the extracellular Fab yield can be maximized by cultivation in the fed-batch medium with complex nutrient supplementation under moderately oxygen limited conditions. Both *E. coli* BL21 and *E. coli* RV308 are good hosts for the extracellular production in the fed-batch medium. However, maximum Fab accumulation in the culture medium requires long cultivation periods during which cell lysis takes place to a significant degree, resulting in presence of background cellular proteins in the medium. In this regard *E. coli* RV308 seems to be a convenient strain for production of extracellular antibody fragments, as it has lower lysis rate than *E. coli* BL21 but under sufficiently oxygen-limited conditions can release substantial amounts of Fab into the cultivation medium in a lysis-independent manner. In both strains, however, maximum recovery of extracellular product while minimizing release of cytoplasmic proteins is a matter of optimizing the harvest time. In some specific cases it may be preferable to collect Fabs from the periplasm, and the best strategy for maximizing the periplasmic yield seems to be expression in *E. coli* RV308 and the fed-batch medium with exclusion of the complex nutrient supplementation at induction. This approach minimizes Fab leakage and maintains higher overall yield than cultivation with the nutrient supplementation under high aeration conditions. Since maximum Fab expression is achieved at relatively low aeration rates, Fab production at larger scale could be well accomplished in vessels such as disposable wave-mixed bioreactors [41] that have lower aeration rates compared to stirred bioreactors. The enzyme-based fed-batch system should be well suited to larger scale Fab production as it has been demonstrated well scalable up to pilot plant scale [42] and applicable to disposable bag bioreactors [43].

Our results also highlight the importance of aeration rate as a cultivation parameter in laboratory-scale shaken cultures which are often performed without appropriate consideration of oxygen transfer. If the aeration efficiency and other factors contributing to oxygen saturation during cultivation are not controlled when the system is scaled up from one type or size of vessel to another, productivity of the culture may vary considerably due to changes in DOT. Changes in aeration can also result in surprising effects beyond expression yield, as was the case with periplasmic protein leakage in this study.

## Conclusions

In conclusion, we demonstrated that the yield and leakage of Fab fragments are highly dependent on expression strain, culture medium, aeration efficiency, and the combination of these parameters. High yields of Fab fragments were obtained in both *E. coli* K-12 strain and BL21 strain in a medium with fed-batch-like glucose feeding, and in *E. coli* BL21 in a glycerol-based autoinduction medium. Regardless of strain and medium, maximum volumetric productivity was achieved under sufficiently oxygen-limited conditions. Also the leakage of Fabs into the culture medium increased considerably under lower aeration conditions. This dependency may cause gaps in reproducibility when scaling up or down if oxygen supply or consumption rate are changed, but it also offers a practical way to efficiently manipulate the ratio of product localization in periplasm and extracellular medium.

## Materials and methods

### Expression constructs

**Figure 7:**
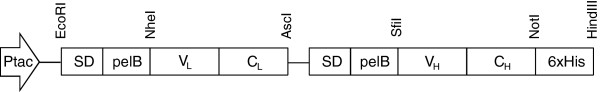
**Schematic presentation of the Fab expression construct.** The Fab fragment is arranged as a bicistronic unit with the light chain (V_L_C_L_) and heavy chain (V_H_C_H_) in different reading frames. Each chain is equipped with *pelB* signal sequence in the N-terminus and the heavy chain is fused with C-terminal hexahistidine tag (6xHis). Ptac: *tac* promoter, SD: Shine-Dalgarno ribosome binding site.

The gene sequences encoding four different Fab fragments (Veijola et al., manuscript in preparation) against N-terminal prohormone of brain natriuretic peptide **(**NTproBNP) were each cloned to a modified pKK233 expression vector backbone (Veijola et al., manuscript in preparation) under the control of *tac* promoter, and transformed into *E. coli* BL21(DE3) and RV308 using standard cloning and transformation procedures. The pKK233 vector encodes resistance to ampicillin. The Fab fragments contained an N-terminal *pelB* signal sequence for periplasmic transport in both the heavy and the light chains. Additionally, a C-terminal hexahistidine tag was included in the heavy chain. Three of the Fab fragments (coded as F1, F16 and F32) bind to their epitopes near the C-terminal end of NTproBNP, while one fragment (coded as 1B10) binds near the N-terminal end of the antigen. The general layout of the expression construct is shown in Figure 7.

### Media

Fed-batch-like cultivation conditions were provided by using the EnBase system with enzyme-based glucose release from soluble polysaccharide. The medium was prepared by dissolving EnPresso® medium tablets (BioSilta, Oulu, Finland) into sterile water. As described previously [22], the medium consists of mineral salts, MgSO_4_, thiamine, trace elements solution, soluble polysaccharide substrate, and a low amount of complex nutrients. After dissolution of the tablets, the medium was supplemented with 1 g l^-1^ glucose and pH was adjusted to 7.4 by adding 1.6 ml of 2M NaOH to each 100 ml of medium. Cultures in shake flasks were supplied with 0.6 U l^-1^ of the glucose-releasing biocatalyst (EnZ I’m, BioSilta) before inoculation. Screening cultures in 24 deep well plates were grown as a batch without biocatalyst until induction. At the time of induction, all cultures in 24 deep well plates and shake flasks were supplied with 3 U l^-1^ biocatalyst and the EnPresso Booster (BioSilta) providing complex nutrients (peptone and yeast extract).

Super Broth medium with MOPS buffering (SB-MOPS) contained (per liter): tryptone 35 g, yeast extract 20 g, NaCl 5 g, MOPS 10 g; pH was adjusted to 7.0. SB-MOPS for pre-induction growth was supplemented with 2 g l^-1^ glucose, and for induction the cells were transferred to fresh glucose-free SB-MOPS.

ZYM-5052 autoinduction medium [20] contained (per liter): tryptone 10 g, yeast extract 5 g, Na_2_HPO_4_ 3.56 g, KH_2_PO_4_ 3.40 g, NH_4_Cl 2.68 g, Na_2_SO_4_ 0.71 g, glycerol 4 ml, glucose 0.5 g, lactose 2 g, trace elements solution 2 ml, and MgSO_4_ 3 mM.

All media contained 100 μg ml^-1^ ampicillin for selective maintenance of the plasmid. For cultivation in the baffled shake flasks media were supplemented with 0.1 ml l^-1^ antifoam (Sigma 204).

### Deep well plate cultivations

Culture media were inoculated with Fab–expressing clones with high cell density glycerol stocks (OD_600_ of 30–70) to OD_600_ of 0.1-0.15. Broth volume was 3 ml in round-bottom square-shaped wells of 24-deep well plates (24dwp; Thomson Instrument, Part No. 931565-G-1X), and the plates were covered with adhesive porous membrane seals (Thomson Instrument, Part No. 899410). All plate cultivations were performed at 250 rpm in an orbital shaker with 25 mm offset (Infors HT Multitron, Infors AG). Under these conditions, the approximate evaporation rate was 7% of original volume within 19 h and 23% within 42 h. The concentration of broth as a result of evaporation as well as the dilution of the fed-batch cultures due to Booster addition were both accounted for when calculating the results so that the evaporation and dilution effects were eliminated from the Fab concentrations.

Cultures in the fed-batch medium were grown overnight at 30°C, followed by induction at 17 h with 0.2 mM IPTG and simultaneous addition of 10x Booster concentrate (to 1:10 v/v) and biocatalyst (3 U l^-1^). Incubation was continued for further 24 h at 30°C.

Cultures in Super Broth medium were grown with 2 g l^-1^ glucose to OD_600_ of 0.5-0.8. 2 × 3 ml cultures were then collected into a single vial, and cells were gently spun down at room temperature. Supernatant was discarded and the pellet was resuspended in 3 ml of glucose-free SB-MOPS with 0.05 mM IPTG (for *E. coli* RV308) or 0.2 mM IPTG (for *E. coli* BL21). IPTG concentrations had been previously optimized for maximum Fab expression in SB-MOPS (data not shown). The suspension was transferred back to 24dwp and incubated for 19 h at 30°C.

Autoinduction cultures in ZYM-5052 medium were incubated for 41 h at 30°C.

### Shake flask cultivations

For flask-scale expression, 50 ml cultures were inoculated with high cell density glycerol stocks to OD_600_ of 0.1-0.15 and incubated in 250 ml baffled Ultra Yield Flasks (UYF; Thomson Instrument, Part No. 931144) covered with adhesive airporous membranes (AirOtop; Thomson Instrument, Part No. 899423). Temperature was 30°C for all flask experiments, and the offset of orbital shaking was 25 mm.

Cultures in fed-batch medium were grown at 250 rpm with 0.6 U l^-1^ of the glucose-releasing biocatalyst for 17 h, and then induced with 0.2 mM IPTG. Together with IPTG, one EnPresso Booster tablet and 3 U l^-1^ of the biocatalyst were added. Shaking speed after induction was alternatively 250 rpm or 150 rpm.

Cultures in autoinduction medium were incubated at 250 rpm or alternatively at 150 rpm.

In all shake flask experiments, broth volume was measured every time a sample was taken. This data was used in the calculation of results to eliminate the effect of different evaporation rates from Fab concentrations and OD data.

### Cultivation with online oxygen monitoring

An additional experiment was performed with the fed-batch medium in a 24 round-well plate with integrated optical oxygen sensors (OxoDish®, PreSens GmbH, Regensburg, Germany) in each well. The plate was placed onto SDR SensorDish® Reader (Presens GmbH), and the plate and reader were fixed to an orbital shaker with 50 mm offset. Cultivations were performed with 1.1. ml culture volume at 30°C and 200 rpm with online recording of dissolved oxygen tension (DOT) in 5 minute intervals. In these experiments Fab fragment F1 was expressed in *E. coli* RV308 in the fed-batch medium. The polysaccharide substrate in the medium was different from the previous experiments, and the peptone component was replaced by an animal-free peptone. Cultivations were started with 0.25 g l^-1^ glucose and varying concentrations of biocatalyst at pH 7.3. Cultures were induced with 0.2 mM IPTG at 18 h, and at the same time half of the cultures received Booster and more biocatalyst (3 U l^-1^). Cultivations were continued for further 24 h after induction.

### Monitoring of culture growth and pH

Culture growth was monitored by offline cell density measurements at selected time points. Cell density was determined by measuring optical density at 600 nm (OD_600_). OD_600_ of 1 corresponds to a dry cell weight of 0.27 g l^-1^. Culture pH level was monitored by offline measurements of 150 μl broth samples by IQ2400 pH probe (IQ Scientific).

### Determination of Fab expression level

To quantitate the Fab yields, 100 μl broth samples were collected and centrifuged at 13,300 × g and 4°C for 4 min. The supernatant was collected into a separate vial, and the cell pellets and supernatants were both stored at −20°C. For cell disruption the pellets were thawed, suspended in 100 μl of BugBuster (Novagen) and lysed by addition of 2 μl Lysonase Bioprocessing Reagent (Novagen). Cell lysates and broth supernatants were prepared for analysis by centrifugation at 13,300 × g and 4°C for 4 min to remove cell debris and other insolubles.

The quantity of functional Fab in the cell lysates and broth supernatants was determined by indirect ELISA. Immuno™ 96-well MaxiSorp™ plates (Nunc) were coated with 0.1 ml of 1 μg ml^-1^ thioredoxin-NTproBNP fusion antigen at 4°C overnight. The wells were washed three times with PBS + 0.05% Tween-20 (PBST), blocked for 20 min with 1% bovine serum albumin and 0.2% gelatine in PBST buffer (BSA-gelatine-PBST), and washed again three times. 0.1 ml of 1:1000 sample dilutions in BSA-gelatine-PBST were added to the wells and left to bind for 1 h at room temperature, followed by eight wash cycles with PBST. Goat anti-mouse IgG (Fab specific) – alkaline phosphatase (Sigma Aldrich) was diluted 1:5000 in BSA-gelatine-PBST and applied as the secondary antibody. The secondary antibody was incubated in the wells for 30–50 min, followed by seven wash cycles. To detect alkaline phosphatase activity, 0.1 ml of p-nitrophenyl phosphate solution (prepared from SIGMA*FAST*™ tablets, Sigma Aldrich) was added to the wells, and the absorbance at 405 nm was recorded with Thermo MultiSkan plate reader after 5 to 45 min depending on the signal strength. To convert the A_405_ signal to Fab concentration in mg l^-1^, purified Fab standards of known concentration were added to the plate to create a standard curve for A_405_ against mg l^-1^ Fab. For each of the four Fabs, the standard curve was created with a purified solution of exactly the same Fab as the binding affinities to the antigen varied widely between the different Fabs.

The periplasmic Fab fraction was analysed from whole cell lysate with the assumption that all detected Fab activity originated from the periplasmic space. It is assumed that only correctly folded and biologically active Fab fragments bound to the antigen and were quantified, and folding of the Fab to its functional form within cytoplasm is generally a very limited occurrence due to the unfavorable redox conditions. Cytoplasmic assembly to functional conformation would be virtually impossible also due to the signal peptide that is only cleaved during translocation to the periplasm. On these grounds all Fab activity in the lysate can be accounted to periplasmic Fab. This was also experimentally verified with a limited number of cell pellet samples by comparing the Fab activity in whole cell lysate and periplasmic extract generated via lysozyme treatment in cold sucrose solution (30 mM Tris–HCl, 1 mM EDTA, 40% sucrose, pH 8.0; Neu and Heppel [44]). Two hours incubation in the lysozyme-sucrose solution at +4°C was sufficient to release periplasmic proteins without lysing the cells. Analysis by ELISA confirmed equal yields of functional Fab in the lysate and the periplasmic extract (data not shown).

### Estimation of cell lysis

To estimate the degree of cell lysis in shake flask cultures, the amounts of total protein in cell pellet and culture supernatant were determined by standard Bradford microplate assay. Dilutions of bovine serum albumin were used to create a linear standard curve. 1:100 dilution of samples was sufficient to measure A_595_ in the linear range, and at this dilution the background absorbance by medium components in supernatant samples was negligible.

## Abbreviations

24dwp: 24 deep well plate; DOT: Dissolved oxygen tension; EB: EnBase; IPTG: Isopropyl β-D-1-thiogalactopyranoside; kLa: Volumetric oxygen transfer coefficient; NTproBNP: N-terminal prohormone of brain natriuretic peptide; OUR: Oxygen uptake rate; PBS: Phosphate buffered saline; SB: Super Broth; UYF: Ultra Yield Flask; ZYM: ZYM-5052 autoinduction medium.

## Competing interests

PN is a co-founder and minor shareholder of BioSilta Oy. PN and AV are inventors in patent applications EP2226380 A1 and EP2356212 A2. KU and JV declare no competing interests.

## Authors’ contributions

KU designed and carried out all cultivations and analyses and wrote the manuscript. JV constructed the expression vectors, participated in the design of the study and revised the manuscript. AV participated in the design of the study and revised the manuscript. PN supervised the study, participated in its design and data analysis and revised the manuscript. All authors read and accepted the final manuscript.

## Supplementary Material

Additional file 1 Standard deviations of ELISA analysis and percentages of extracellular Fab F1 in the cultivations with online DOT monitoring. Click here for file

Additional file 2 Standard deviations of ELISA analysis, percentages of extracellular Fab and estimates for cell lysis in shake flask cultivations. Click here for file

Additional file 3 Medium pH in shake flask cultivations. Click here for file

Additional file 4 Standard deviations of ELISA analysis and percentages of extracellular Fab F1 in small-scale cultivations with varying culture volumes. Click here for file
